# Sustainable Upcycling of Spent Electric Vehicle Anodes into Solution-Processable Graphene Nanomaterials

**DOI:** 10.1021/acs.iecr.2c02634

**Published:** 2022-10-28

**Authors:** Jason Stafford, Emma Kendrick

**Affiliations:** ^†^School of Engineering and ^‡^School of Metallurgy & Materials, University of Birmingham, BirminghamB15 2TT, U.K.

## Abstract

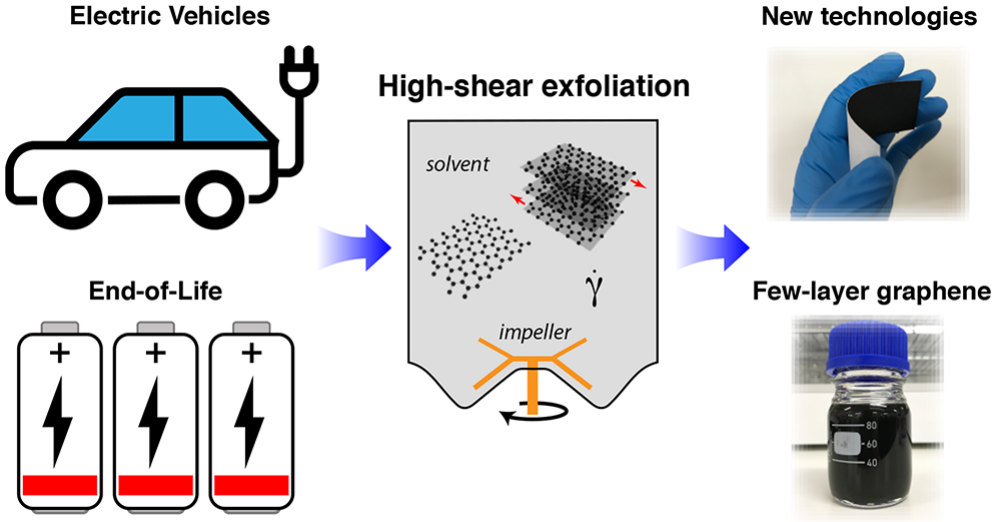

A major transition to electric vehicles (EVs) is underway globally, as countries target reductions in greenhouse gas emissions from the transport sector. As this rapid growth continues, significant challenges remain around how to sustainably manage the accompanying large volumes of waste from end-of-life lithium-ion batteries that contain valuable rare earth and critical materials. Here, we show that high-shear exfoliation in aqueous surfactants can upcycle spent graphite anodes recovered from an EV into few-layer graphene dispersions. For the same hydrodynamic conditions, we report a process yield that is 37.5% higher when using spent graphite anodes as the precursor material over high-purity graphite flakes. When the surfactant concentration is increased, the average atomic layer number reduces in a similar way to that of high-purity precursors. We find that the electrical conductance of few-layer graphene produced using the graphite flake precursor is superior and identify the limitations when using aqueous surfactant solutions as the exfoliation medium for spent graphite anode material. Using these nontoxic solution-processable nanomaterial dispersions, functional paper-based electronic circuit boards were fabricated, illustrating the potential for end-to-end, environmentally sustainable upcycling of spent EV anodes into new technologies.

## Introduction

**Figure 1:**
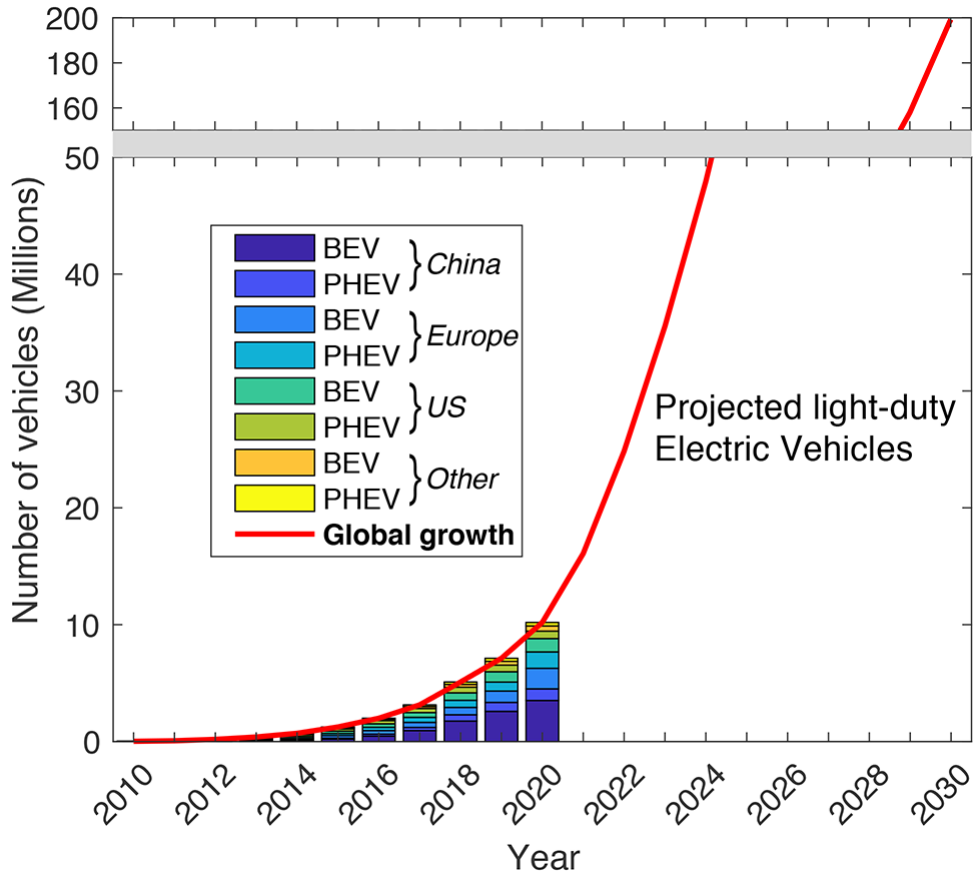
Historical and projected growth in battery electric vehicles (BEV) and plug-in hybrid electric vehicles (PHEV) from 2010 to 2030.^11^ Based on IEA data from the IEA (2021) Global EV Data Explorer, www.iea.org/statistics, all rights reserved, as modified by J. Stafford.

Electric vehicles (EVs) have emerged as the primary solution for addressing greenhouse gas emissions in transport, particularly for light-duty vehicles. In the past decade, the number of EVs on the road has grown from almost negligible numbers to ∼10 million as the urgency for climate action has been recognized by governments and manufacturers globally. Over the next 10 years, this is projected to increase dramatically from ∼10 to 200 million (Figure 1). Both battery electric vehicles (BEV) and plug-in hybrid electric vehicles (PHEV) currently utilize lithium-ion batteries to store and deploy electrical energy. This remarkable rate of EV adoption poses a major waste management challenge as lithium-ion batteries reach their end of life.^1^

Current material recovery processes are insufficient, and there are opportunities to improve the sustainability of the entire battery manufacturing lifecycle.^1^ Pyrometallurgy and hydrometallurgy recovery is restricted to high-value materials such as cobalt, lithium, nickel, copper, and aluminum with graphite anodes seen as having a low recovery value. Graphite is more abundant, although natural resources are geographically concentrated. A reliance on importation has lifted it to the status of strategic importance and on critical minerals lists for many nations and regions (e.g., UK, EU). Despite being placed on the critical minerals list and contributing to 20% of the total lithium-ion battery weight,^2^ graphite is currently burned as an energy source, used as a reducing agent in pyrometallurgy, and ultimately disposed of as waste. As the demand for graphite in lithium-ion battery applications increases to meet the global growth of EVs, developing solutions for a circular materials system will be crucial for the environment and economic security.

Developing effective methods that recycle graphite anodes for reuse in new batteries has recently become a focus area for addressing this waste problem.^3−5^ A complementary option that will be explored in this work is the upcycling of graphite anodes into graphitic nanomaterials that could have multiple end uses. For example, Large et al. used size-selected graphene nanosheets to print radio frequency antennas and thin-film electronics.^6^ Using melt mixing, Paton et al. dispersed small volume fractions (0.07 wt %) of shear exfoliated graphene nanosheets in poly(ethylene terephthalate) (PET), improving the strength of PET by 40%.^7^ Graphene nanosheets can also be used in 2D/2D heterostructures for the improvement of visible-light photocatalytic water treatment processes by enhancing the separation of charge carriers from the parent semiconducting photocatalyst.^8^

Graphite is a layered material containing individual graphene layers held together by van der Waals forces. Various chemical and nonoxidizing top-down liquid exfoliation processes have been developed to synthesize few-layer graphene and other graphene-related materials from graphite.^8−10^ By taking advantage of synthesis methods in the field of two-dimensional materials, it may be possible to develop scalable upcycling processes to handle the large waste streams from end-of-life EV batteries.

Liquid-phase exfoliation techniques have recently been applied to anode graphite recovered from lithium-ion batteries in electronic devices to synthesize graphene, graphene oxide (GO), and reduced graphene oxide (rGO). The techniques vary from ultrasonication-assisted,^12^ mixed chemical–mechanical approaches,^13^ and entirely chemical exfoliation methods.^14−16^ However, challenges remain, particularly around the environmental sustainability of processes that can convert lithium-ion anodes to graphene, GO, and rGO materials.^17^

Previous approaches have required the use of either toxic solvents (e.g., NMP) in the treatment/pretreatment step or methods that were based on other chemical processes (e.g., modified Hummer’s method). In this work, we explore the use of high-shear exfoliation, without harsh chemicals or toxic solvents, to produce solution-processable graphene nanomaterials that are then used to fabricate paper-based electronic devices. We assess this upcycling approach on anode graphite recovered from an electric vehicle whose battery has reached its end of life. By doing so, the methods and research outcomes are directly relevant to this rapidly growing industrial sector.

## Methods

### Electric Vehicle Graphite Recovery

**Figure 2:**
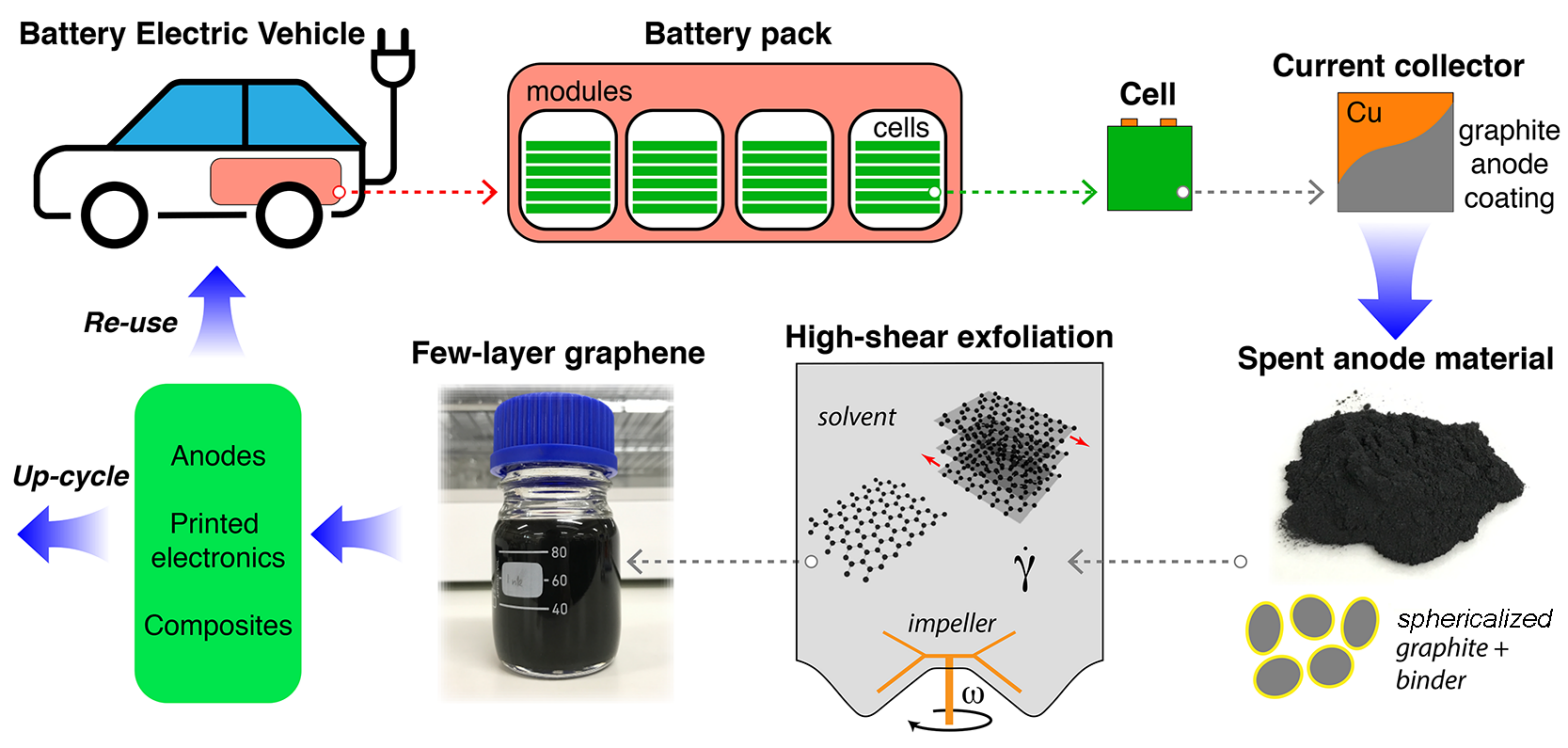
Illustration of the graphite anode recovery and conversion process. High-shear exfoliation in liquids is applied to convert the waste graphite material into few-layer graphene dispersions that can be either reused as battery anode material or upcycled for other technologies.

Spent graphite anode material was reclaimed from a Nissan Leaf (generation 1). An illustration of the battery breakdown to the cell level is shown in Figure 2. Graphite was recovered from the copper current collector using a cell teardown process described by Marshall et al.^18^ The vehicle battery was considered to be at its end of life (2.5 V) and was discharged to a 0% state of charge. The resulting anode black mass powder contained the graphite material and a binder (poly(vinylidene fluoride), PVDF) with an estimated content of <5 wt %. A volume-based median particle size of 21 μm was measured using laser diffraction (Malvern Mastersizer). Previous observations using scanning electron microscopy confirm a large number of particles with diameters of ∼20 μm within the anode black mass.^18^

### Synthesis of Few-Layer Graphene

Graphite recovered from electric vehicle anodes was dispersed in an aqueous-surfactant solution (*V* = 160 mL) using deionized water (5 MΩ m) and sodium cholate (Sigma-Aldrich, C1254). The binder was retained in the starting graphite material to investigate synthesis without any pretreatment steps that require toxic solvents such as NMP to wash PVDF from the graphite. The dispersion was subjected to high-shear exfoliation using a cylindrical stirred vessel with diameter 70 mm, height 95 mm, and a contoured base (Figure 2). A four-bladed impeller with diameter *D* = 55 mm was rotated at a speed of ω = 20 000 ± 1500 rpm (350W, Kenwood BLP31), resulting in turbulent flow inside the vessel (*Re* = *ρωD*^2^/μ ≈ 10^6^) and shear rates of  s^–1^.^19^ This rotational speed was chosen to operate above the critical criterion required to exfoliate few-layer graphene from graphite flakes,  s^–1^.^7^

To avoid overheating the motor and the liquid dispersion, the impeller was rotated for 1 min and then turned off for 5 min. During the off period, the vessel was surrounded by ice and placed in a container inside a freezer at −20 °C. This ensured that the dispersion was kept at ambient room temperature for the beginning of each 1 min process interval. A total of 15 process intervals were conducted, resulting in an overall process time of *t*_ex_ = 15 min for each material synthesis performed.

Finally, the performance of the shear exfoliation upcycling process for spent EV anode materials was assessed by conducting an equivalent set of experiments on high-purity graphite as a benchmark. Graphite flakes (Sigma-Aldrich, 332461) were chosen because they are one of the most commonly used precursor materials for the production of few-layer graphene in the literature. Laser diffraction measurements were taken, and these flakes were found to have a volume-based median particle size of 550 μm. Identical material preparation, high-shear exfoliation, and postproduction steps were followed as described for spent EV anode graphite in the Methods section.

### Material Characterization

After liquid-phase exfoliation, the aqueous-surfactant dispersions were pipetted into centrifugation tubes with a capacity of 15 mL and centrifuged at a relative centrifugal force (RCF) of 243*g* for 45 min. The top 5 mL of the supernatant contained few-layer graphene and was removed for analysis using UV–vis–nIR spectroscopy (PerkinElmer 365). The average atomic layer number (*N*) was obtained by measuring the extinction spectra (*E*(λ)) of the nanomaterial dispersions and utilizing spectroscopic metrics for graphene, *N* = 25(*E*_550 nm_/*E*_max_) – 4.2.^20^

The few-layer graphene concentration (*C*_gr_) was determined by filtering the dispersions through 25-mm-diameter PTFE filters with a pore size of 220 nm. The mass of few-layer graphene retained on the filters was measured and then used to calculate the extinction coefficients, ε(λ). At λ = 660 nm, the extinction coefficient is independent of the nanosheet thickness and size. Therefore, this wavelength was chosen when measuring the concentration of the few-layer graphene dispersions using the Lambert–Beer relationship, *C*_gr_ = *E*_660 nm_/ε_660 nm_*L*, where *L* is the optical path length of the cuvette (10 mm). The extinction coefficient was measured to be ε_660 nm_ = 1014 L g^–1^ m^–1^ for material produced using the spent EV anode precursor and ε_660 nm_ = 1521 L g^–1^ m^–1^ for the material produced using the graphite flake precursor.

Although there are variations in the values of extinction coefficients in the literature, the value obtained for few-layer graphene exfoliated from high-purity graphite flakes is in close agreement with previous work on liquid exfoliation of the same precursor in aqueous-surfactant solutions.^21^ The lower value for the extinction coefficient of graphene derived from the spent EV anode precursor may be attributed to the differences in quality of the starting graphite sources. For example, material dependencies were shown recently to have a non-negligible effect on the optical properties of graphene flakes in aqueous solutions.^22^ Our findings demonstrate that measurements of extinction coefficients are important to consider when testing different graphite sources, even under identical liquid exfoliation conditions and solvents.

## Results and Discussion

### High-Shear Upcycling Performance

**Figure 3:**
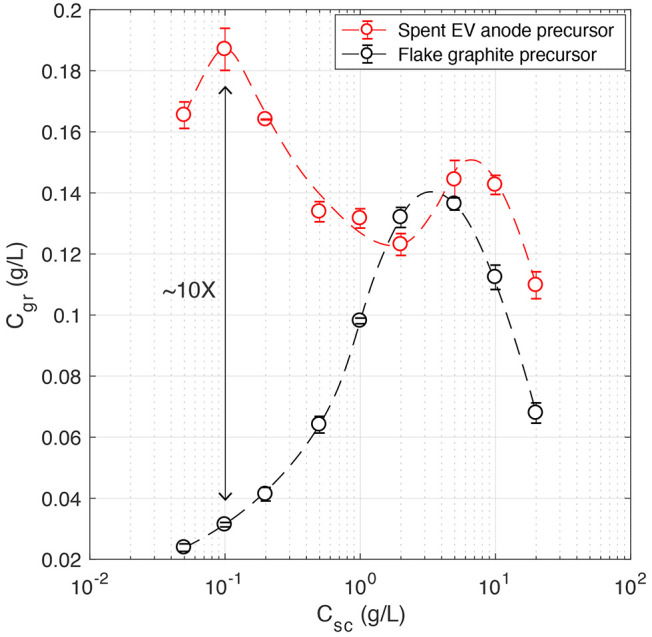
Few-layer graphene concentration produced from spent EV anode material and flake graphite material precursors in aqueous-surfactant solutions across a range of surfactant concentrations. The impeller rotational speed, processing time, and initial graphite concentration were ω ≈ 20 000 rpm, *t*_ex_ = 15 min, and *C*_*i*_ = 10 g/L, respectively.

**Figure 4:**
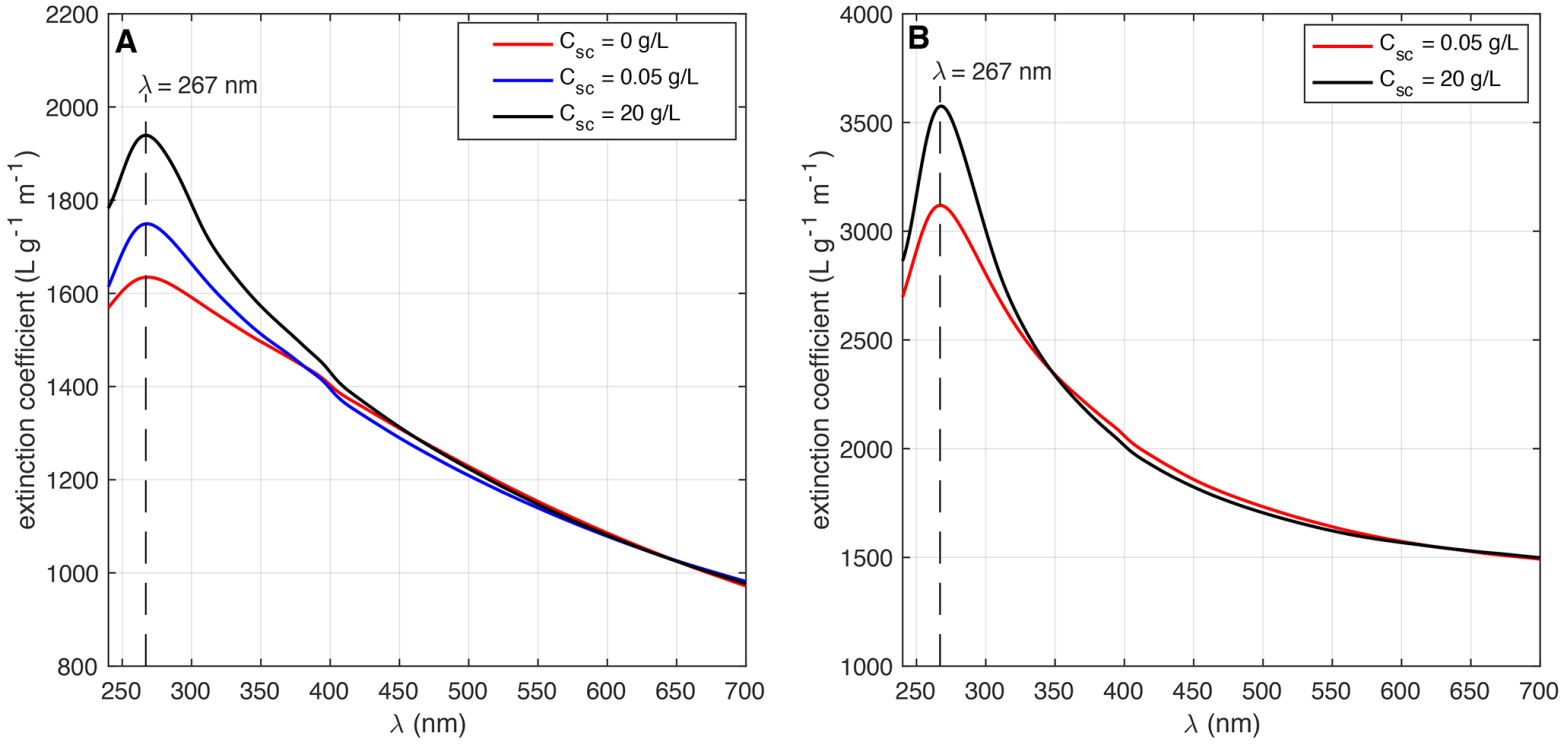
UV–visible optical extinction spectra of few-layer graphene dispersions produced using (A) a spent EV graphite anode and (B) flake graphite precursors. ω ≈ 20 000 rpm, *t*_ex_ = 15 min, and *C*_*i*_ = 10 g/L.

The upcycling performance of high-shear exfoliation in aqueous surfactants was assessed by comparing the production outputs of the spent graphite anode and high-purity graphite flake precursors. The concentration of few-layer graphene products for a range of surfactant concentrations (*C*_sc_ ≈ 10^–1^–10^1^ g/L) is shown in Figure 3. In general, across the range of sodium cholate concentrations explored, the product concentration was higher when using the spent EV graphite anode as a precursor. This was most notable for lower surfactant concentrations (0.05 ≤ *C*_sc_ ≤ 1 g/L) where differences of up to 10-fold were found. For surfactant concentrations of 1 ≤ *C*_sc_ ≤ 20 g/L, the concentration of few-layer graphene extracted from the spent anode precursor was comparable to and up to twofold higher than that of the graphite flake precursor. The optical extinction spectra for both materials and various surfactant concentrations are shown in Figure 4. Both products were found to have an absorption peak at λ = 267 nm, indicative of the electronic conjugation for graphene.^23^

The maximum yields (*C*_gr_/*C*_*i*_) after 15 min of high-shear exfoliation ( s^–1^) were 1.87 wt % (upcycled EV anode) and 1.36 wt % (graphite flake), respectively. Although the absolute yields are low, they are favorable when compared to other scalable nonoxidizing liquid exfoliation techniques such as sonication,^7^ microfluidization,^24^ high-shear mixers,^7^ spinning disc, and Taylor–Couette-type approaches^25^ that produce yields of 0.1–3 wt % for longer processing times of ∼1–10 h. Furthermore, we took unexfoliated EV anode sediment, redispersed it in fresh deionized water/sodium cholate solution (*V* = 160 mL), and repeated the shear exfoliation process to explore if it is possible to extract additional graphene nanomaterial using solvent exchange. This second exfoliation step produced 69% of the initial output over the same 15 min process interval, demonstrating that graphite anode sediment can be reprocessed multiple times and that yields of >3% can be obtained in only 30 min.

We can use the process yield results to estimate the upcycling material production rate. Using *C*_*i*_ = 100 g/L and *V* = 0.16 L, a production rate of ∼1.2 g/h could be achieved using this high-shear approach. This also compares favorably with other batch shear exfoliation methods. For example, high-shear mixers operating in batch mode can deliver production rates of ∼5.3 g/h for *C*_*i*_ = 100 g/L and *V* = 300 L.^7^ Although the absolute production rate is higher for the latter, the difference in the volume of input resources (water, graphite, and surfactant) and process waste is substantially larger by ∼10^3^. Alternatively, we can investigate the scale required to achieve the production rates of a batch high-shear mixer. For a similar high-shear stirred vessel in this work, only with larger process volumes of up to ∼1 L, Pérez-Álvarez et al. showed that few-layer graphene concentration follows the scaling relationship , above the critical exfoliation criteria.^19^ Applying this scaling, we estimate an upcycling production rate of ∼5 g/h with a process volume of *V* = 3 L. This is only 1% of the volume required by high-shear mixing in batch operation, indicating that high-shear upcycling of spent graphite anodes is a promising and environmentally sustainable approach.

In terms of end-use considerations, it is equally significant that the maximum yield using a spent graphite anode was obtained for a surfactant concentration that was one order of magnitude lower than for graphite flakes. Indeed, we also measured the concentration for high-shear upcycling in water only (*C*_sc_ = 0), and this resulted in a yield of 0.69 wt %. Considering that residual surfactant can adversely impact material properties and can be challenging to remove from solution-processed functional devices (e.g., requiring high temperature annealing^6^), high-shear upcycling has the potential to provide the sustainable synthesis of few-layer graphene with low additive requirements.

Interestingly, two different characteristics were observed for each precursor. The upcycled product featured a bimodal concentration profile, whereas the few-layer graphene produced from graphite flakes contained a single peak in concentration at *C*_sc_ ≈ 4 g/L. The occurrence of a single peak in concentration can also be seen in previous studies on the liquid exfoliation of graphite in aqueous-surfactant solutions using sonication^21^ and high-shear exfoliation.^19,26^ Recently, using WS_2_ as a model system, Griffin et al. showed that a falloff in nanosheet concentration occurs at ∼10 mM for all ionic surfactants.^27^ The authors measured reductions in ionic conductivity of the aqueous-surfactant dispersions in this region and hypothesized this behavior to be due to electrostatic screening. The sharp decrease in few-layer graphene concentration shown in our work for *C*_sc_ > 5 g/L coincides with *C*_sc_ ≈ 10 mM. The few-layer graphene produced by upcycling also follows a similar reduction in concentration in this region (*C*_sc_ ≈ 10 mM). This suggests that the same nanosheet destabilizing mechanism at *C*_sc_ ≈ 10 mM also applies to the solution processing of spent graphite anode materials in aqueous surfactants.

In the low-surfactant-concentration region (0.05 ≤ *C*_sc_ ≤ 1 g/L), the primary (largest) peak in *C*_gr_ for the upcycling process occurs (*C*_sc_ = 0.1 g/L). This contrasts with the trend observed for shear exfoliated high-purity graphite flakes, where the dispersions have poor stability and the lowest few-layer graphene concentrations. Further investigation into this finding is necessary; however, the enhanced concentrations using spent anode precursors may be attributed to (1) the presence of significantly more edge sites on the sphericalized particles for intercalation and delamination to occur; (2) graphite expansion from the intercalation of lithium between layers during charge/discharge battery cycles; and (3) increased wettability of the PVDF binder due to the presence of the surfactant.

Expanding on the previous points (1–3), the sphericalized graphite particles from the EV anode are much smaller than the flake graphite particles, with a D50 of 21 μm compared to 550 μm. As part of the sphericalization process, these particles are jet milled to create a morphology which also contains more sites for the intercalation and delamination of layers than the graphite flakes. Also, during the operational lifetime of the battery, lithium ions intercalate into the graphitic layers in the anode, weakening the van der Waals attractive force. It has been suggested that this process can expand the layer spacing by an average of 3.5% (0.352 nm) and up to 14.7% (0.39 nm).^13^ The chemical and thermal expansion of graphite as a pretreatment step in the synthesis of graphene and graphene-derived materials (e.g., GO and rGO) is a method known to improve the production output;^10^ therefore, the presence of dilated layers in the anode graphite would also benefit the exfoliation process. Finally, the PVDF binder is hydrophobic, which should reduce the dispersibility of graphitic particles in water. However, anionic surfactants have been shown to have an an affinity for PVDF and increase the wettability below the critical micelle concentration (CMC).^28^ Increased wettability may also play a role in the enhanced anode material dispersibility that is observed here below the CMC for sodium cholate. Nevertheless, the high recovery performance is encouraging, and further research in this low surfactant region would help to optimize the mechanisms which enhance concentration using spent graphite anode material.

**Figure 5:**
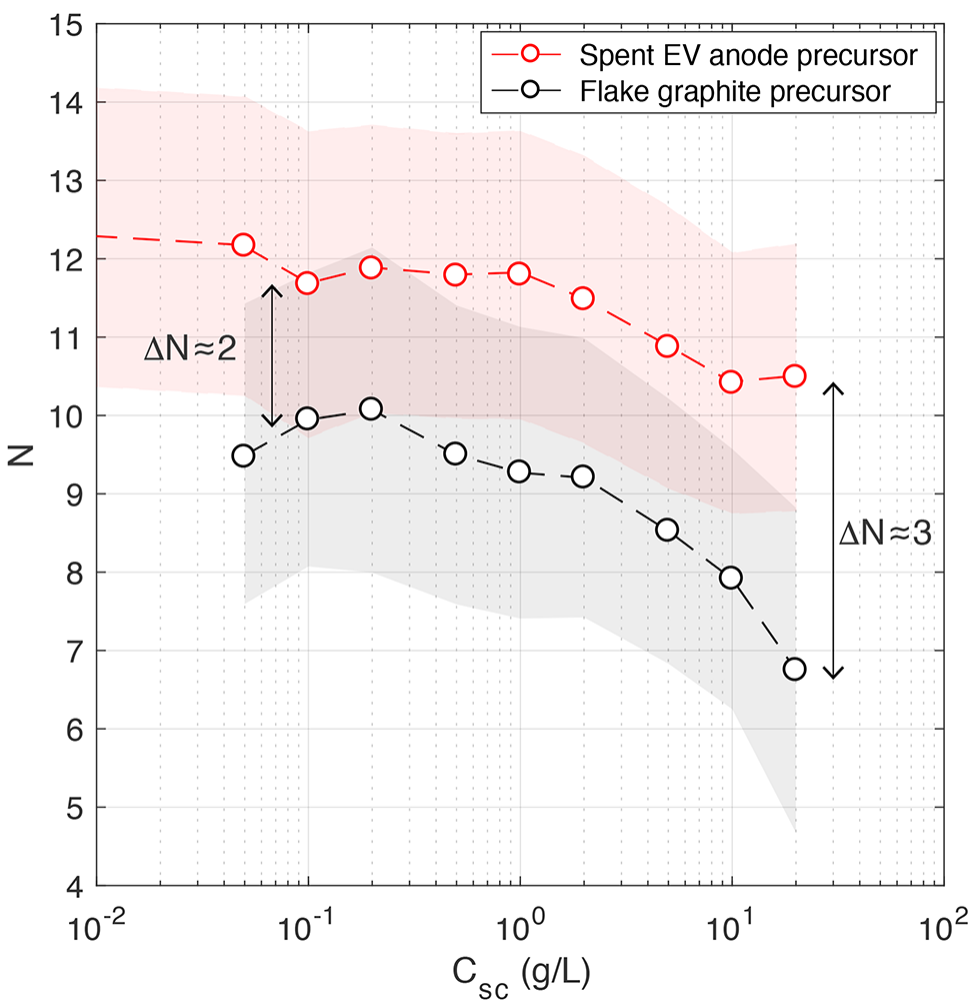
Variation in the average number of atomic layers with surfactant concentration for few-layer graphene dispersions produced using a spent EV graphite anode and flake graphite precursors. ω ≈ 20 000 rpm, *t*_ex_ = 15 min, and *C*_*i*_ = 10 g/L.

Variations in surfactant concentration modified the extinction spectra for both graphene materials in the UV wavelength region (Figure 4). For few-layer graphene, this change in shape is a signature of a change in the average number of atomic layers.^20^ The number of atomic layers is plotted against surfactant concentration in Figure 5, and a similar trend was observed for both few-layer graphene materials. Thicker sheets are present in low surfactant concentration dispersions. The thickness remains relatively constant up to *C*_sc_ ≈ 1 g/L and then decreases further as the surfactant concentration increases. This corresponds to the threshold of *C*_sc_ ≈ 10 mM and aligns with the previous observations for *C*_gr_ noted above and for other 2D materials synthesized from high-purity precursors.^27^ This demonstrates that the atomic layer number of few-layer graphene exfoliated from spent graphite anodes recovered from EVs can also be tuned using surfactants.

The rate of change in the layer number with *C*_sc_ was found to be lower for the upcycled material. At the highest *C*_sc_ = 20 g/L, the differences in the layer number increased from Δ*N* ≈ 2 to 3. Overall, it appears that few-layer graphene dispersions produced from graphite flakes have a lower average number of layers than the upcycled product. This suggests that for the same hydrodynamic conditions and aqueous-surfactant solutions, the quantity of few-layer nanosheets produced from spent EV anodes is less than for the high-purity graphite flakes. Shear exfoliation using turbulent flows erodes the outer surfaces of graphite flakes to produce few-layer graphene.^25^ The resulting few-layer sheets continue to decrease in thickness by rate-controlling processes that span the entire range of turbulent energy-containing flow structures down to the Kolmogorov length. The binder layer that coats the surfaces of graphite anode particles and smaller exfoliated platelets potentially acts as a barrier for slip and peel mechanisms of delamination to proceed at the nanoscale.

The spectroscopic metric used to calculate *N* is based on an empirical correlation that predicts the average layer number to within 15% for a number of different graphite precursors.^20^ The shaded regions in Figure 5 represent the combined correlation and experimental uncertainties. The bounds of these uncertainties in the average layer number for the different precursor materials overlap, and an analysis focusing on individual nanosheet size distributions would ultimately confirm the thickness statistics (e.g., using AFM). However, these differences in material quality (thickness) are in agreement with measurements of electronic properties presented in the following section.

### Application to Paper Electronics

**Figure 6:**
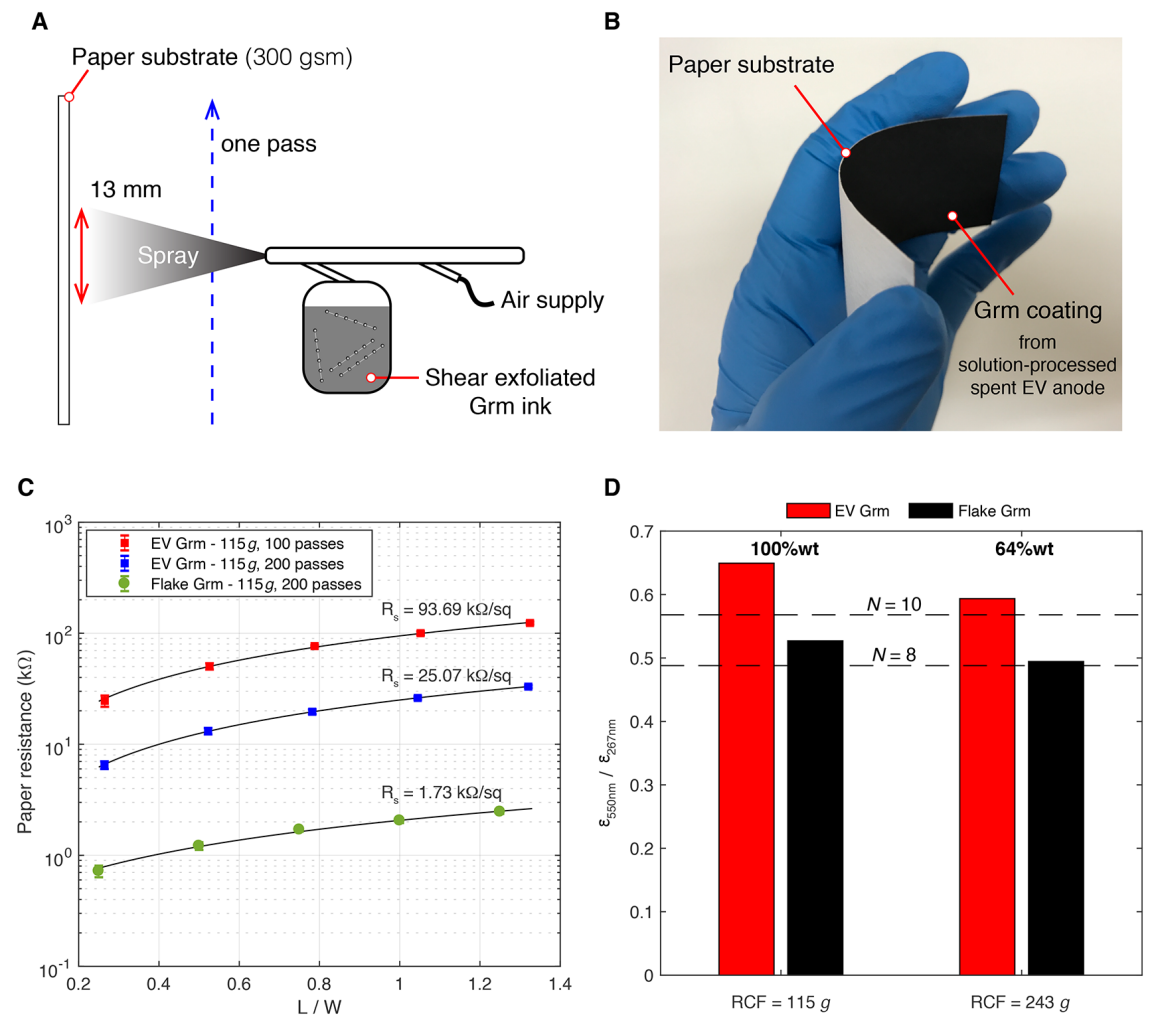
Performance of upcycled EV anode material. (A) Schematic of thin film deposition of graphene-related nanoplatelet materials (Grm) onto a paper substrate using airbrushing. (B) Image of a Grm coating produced from shear exfoliated spent graphite anode material and 100 spray passes. (C) Electrical resistance of various Grm coatings spray deposited onto paper. (D) Comparison between the spectroscopic ratio ε_550 nm_/ε_267 nm_ for EV Grm and flake Grm dispersions, with the ratios corresponding to average layer numbers of 8 and 10 highlighted. The same Grm concentration was used for all inks during airbrushing (*C*_Grm_ = 1.66 g/L). These were prepared using *C*_*i*_/*C*_sc_ = 100 (EV), *C*_*i*_/*C*_sc_ = 20 (flake), ω ≈ 20 000 rpm for *t*_ex_ = 25 min, and RCF = 115*g* for 20 min.

Maintaining the theme of environmentally compatible processes, we explored the application of upcycled graphene materials for fabricating paper printed circuit boards. The electronic properties were investigated by solution-processing graphene dispersions to create paper-based conductive thin films. Graphene inks were prepared using shear exfoliation in deionized water/sodium cholate followed by centrifugation and spray deposition onto paper substrates (100% cellulose, acid-free, 300 gsm, cold-pressed). An illustration of the spray deposition process is shown in Figure 6A.

With few-layer graphene yields on the order of wt after a 15 min exfoliation processing time, the mass of the remaining graphite is large. However, the number of graphite particles retained in noncentrifuged liquid-phase exfoliated dispersions has been found to be low (∼5.5%) compared to few-layer graphene nanosheets with *N* ≤ 10.^29^ This suggests that the graphite mass is dominated by a small number of poorly exfoliated particles. To remove these, the as-prepared dispersions were centrifuged at RCF = 115*g* for 20 min, following a similar approach recently described for biocompatible few-layer graphene inks.^30^ As these formulations contain thicker nanoplatelets, we refer to the inks as graphene-related material (Grm) to distinguish them from the few-layer graphene dispersions formulated at RCF = 243*g*.

The ink concentration was increased to above 1 g/L by exfoliating spent graphite anode material at *C*_*i*_ = 50 g/L and using a surfactant concentration, *C*_*i*_/*C*_sc_ = 100, corresponding to the peak *C*_gr_ shown in Figure 3. This resulted in an ink concentration of *C*_Grm_ = 1.66 g/L. To obtain an equivalent ink concentration from the high-purity graphite flake precursor, exfoliation was first performed using *C*_*i*_ = 100 g/L and *C*_*i*_/*C*_sc_ = 20, resulting in *C*_Grm_ = 2.8 g/L. This “flake Grm” product was then diluted to match the concentration of the upcycled “EV Grm” product before spraying the paper substrate.

A 90 mm × 40 mm area was airbrushed with a supply pressure of 20 psi, resulting in a graphene material deposit of 18.4 g/m^2^ per spray pass. Multiple spray passes were used to create flexible paper substrates that were electrically conducting on the coated side and insulating on the other. The coatings were physically robust with strong adherence to the paper backing and the ability to withstand bending and folding. To accelerate the drying process, air at a temperature of 200 °C was blown over the paper using a heat gun, evaporating the water solvent after each spray pass. An example of a paper substrate with 100 passes of a shear exfoliated graphite anode material is shown in Figure 6B. Assuming negligible porosity and uniform thickness, the minimum thickness limit of the dried nanoplatelet film can be estimated to be ∼8 μm for 100 spray passes. However, some porosity is likely to exist, and the actual film thickness will be larger and contain variability from the coating method. Although the cold-pressed paper substrate has visible roughness that can contribute to this variability, it also provides increased substrate porosity (over that of hot-pressed paper substrates) that is advantageous for wetting and nanoplatelet film reproducibility.^31^ This is confirmed by the low-magnitude error bars in Figure 6C which include the standard deviation in electrical resistance for different nanoplatelet films tested. The variation in the electrical resistance was found to be below 12% for all Grm coatings.

The sheet resistances of various spray-coated paper substrates were measured in accordance with the transmission line method.^6^ Copper tape electrodes (75 μm thickness, 6 mm width) were positioned in parallel and bonded to the Grm coated paper (*W* = 40 mm) using low contact resistance adhesion (*R* = 0.001Ω). The electrical resistance was measured between electrodes spaced various distances, *L*, apart (Keithley model 2000). The sheet resistance was then determined from the slope of the linear trend between *L*/*W* and paper resistance, shown in Figure 6C. The sheet resistances span ∼1–10 kΩ/square, which is similar to that observed in other studies investigating spray-coated graphene inks on paper substrates.^31^

Using the flake Grm as the reference, the sheet resistance was found to be 10-fold higher for the EV Grm coating. This is due to a combination of factors including thicker and smaller nanosheets, the presence of a residual anode binder in the nanosheet network, and the potential presence of nanosheet defects. Under the same shear exfoliation conditions, Figure 5 shows that the number of layers in the upcycled graphene dispersions is greater than when using high-purity graphite flakes (by Δ*N* ≈ 2 to 3). To explore this further, we isolated the fraction of few-layer graphene contained in the shear exfoliated Grm inks by performing an additional centrifugation step at RCF = 243*g* and 45 min. We measured the extinction spectra for both RCF = 115*g* and RCF = 243*g* and compared the spectroscopic ratio ε_550 nm_/ε_267 nm_ as a proxy to illustrate the changes in the atomic layer number (Figure 6D). The fraction of few-layer graphene (*N* ≈ 10) in the EV Grm ink was found to be ∼64 wt %. In contrast, the graphitic material within the flake Grm ink is dominated by few-layer graphene nanosheets (*N* < 10) including a significant fraction with *N* = 8 (∼64 wt %). This difference in composition impacts the electronic properties of the deposited film and results in a higher sheet resistance for the upcycled spent EV anode material.

The conductance of the thin film produced from EV Grm increases almost fourfold when the number of spray passes is doubled to 200. This highlights that further reductions in sheet resistance toward kΩ/square are possible by continuing to increase the number of spray passes. Of course, this would be at the expense of using more material; however, the maximum yield was 37.5% higher for a spent graphite anode. A sustainable pretreatment step to remove the PVDF binder and additives prior to shear exfoliation would also reduce the sheet resistance for the Grm ink. Furthermore, size selection and surfactant removal have recently been shown to have significant influences on the electrical conductivity (10-fold changes) of graphitic nanoparticulate materials exfoliated in water/Triton X-100 surfactant solutions.^6^ Similar approaches may also work with liquid exfoliated spent graphitic anode materials, maximizing the conductivity of upcycled functional inks where graphitic nanoparticles, additives, and surfactants are present.

**Figure 7:**
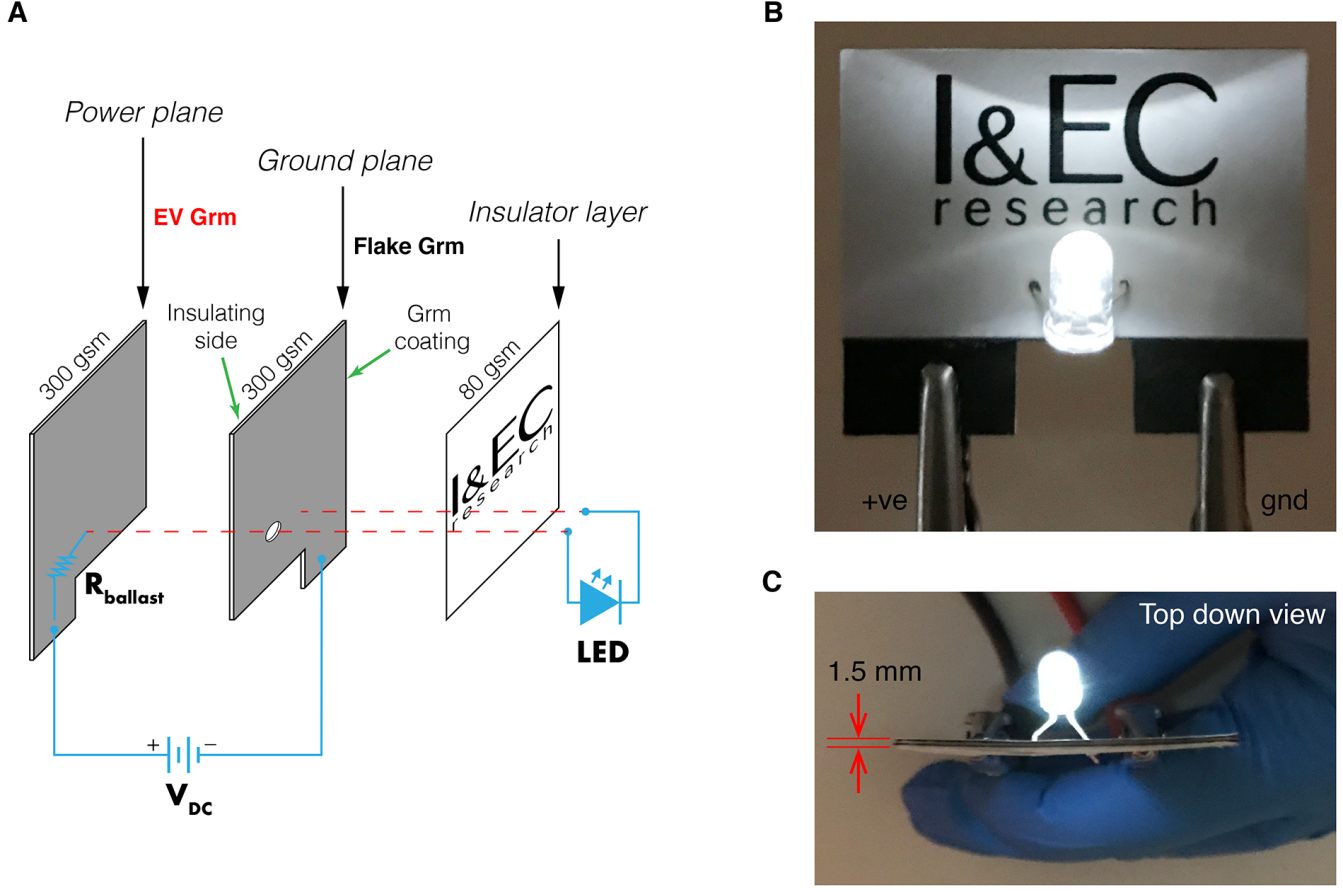
Basic paper circuit board fabricated using shear exfoliated anode material recovered from an end-of-life electric vehicle battery. (A) Paper layers and light-emitting diode (LED) circuit. (B) Functioning electronics powered using a 12 VDC supply. (C) Top down view of the paper circuit board assembly indicating an overall thickness of 1.5 mm.

Although the electrical conductivity of the paper coatings was superior using high-purity graphite flakes, contemporary electronic circuits rely on a suite of different components and materials (conductors, insulators, semiconductors). We exploited the differences in electronic properties between EV Grm and flake Grm solution-processed thin films to fabricate a simple paper circuit board with a white light-emitting diode (Figure 7). Paper substrates were coated using the same approaches described above and illustrated in Figure 6A. The ground plane was coated with flake Grm, and the power plane was coated using upcycled EV Grm ink. The paper substrates were then cut to size and glued together to create insulator–conductor multilayers with an overall circuit board thickness of 1.5 mm. The EV Grm layer provided a dual purpose, acting as the power plane for the +ve LED pin to connect to and as a ballast resistor that prevented an overcurrent when using a 12 VDC supply. This simple example demonstrates that shear exfoliation in aqueous-surfactant solutions can be used as a facile approach for upcycling battery anode waste and contributing to material circularity for electric vehicles.

## Conclusions

This work demonstrates that high-shear exfoliation in aqueous surfactants is a viable approach for upcycling spent graphite anodes from electric vehicles into solution-processable graphene. The nanomaterial yield was found to be comparable to and in most cases higher than that obtained using high-purity graphite flakes as a precursor. The maximum concentration recovered using this upcycling approach was 37.5% higher than the peak concentration of few-layer graphene obtained from graphite flakes. By demonstrating the feasibility of shear exfoliation, this work shows that other shear exfoliation techniques that have achieved high yields using natural graphite precursors (e.g., 10–100 wt %) may also process spent electric vehicle anodes with similarly high yields. However, it is noted that significant improvements to this technique will be necessary to achieve economical upcycling of spent anodes from electric vehicles. Spectroscopic measurements suggest that the average atomic layer number in aqueous-surfactant dispersions is 2 to 3 atomic layers less when using the high-purity graphite flake precursor. The sphericalized graphite morphology together with the presence of the PVDF binder impacts production output, particularly at low surfactant concentrations of *C*_sc_ < 10 mM. At concentrations above this, the surfactant influences the upcycled product concentration and layer number similarly to few-layer graphene exfoliated from graphite flakes. Finally, we investigated the use of upcycled graphene-related material for fabricating paper electronics. The sheet resistance of thin films formed from upcycled material is an order of magnitude higher, which may be addressable using alternative solvents to remove the binder and any additives. However, the environmental sustainability of the proposed water-based liquid processing techniques is advantageous over chemical treatments or traditional toxic solvent use. Furthermore, lithium-ion batteries also utilize different binders that are dissolvable in water (e.g., carboxy methyl cellulose) and graphite sources (e.g., natural, synthetic) which provide opportunities for improvements without sacrificing the sustainability of the high-shear upcycling process.
